# Retrotransposon Silencing During Embryogenesis: *Dicer* Cuts in LINE

**DOI:** 10.1371/journal.pgen.1003944

**Published:** 2013-11-07

**Authors:** Geoffrey J. Faulkner

**Affiliations:** 1 Cancer Biology Program, Mater Medical Research Institute, South Brisbane, Australia; 2 School of Biomedical Sciences, University of Queensland, Brisbane, Australia; Medical Research Council Human Genetics Unit, United Kingdom

Fossilised mobile genetic elements, including Long Interspersed Element-1 (LINE-1 or L1) retrotransposons, comprise at least two-thirds of the human genome [1]. Their molecular history is reminiscent of speciation and natural selection, where, as noted by Carl Sagan, “Extinction is the rule. Survival is the exception” [2]. Broadly, the life cycle of a retrotransposon begins with innovation to evade host genome surveillance, followed by “copy-and-paste” retrotransposition and, finally, quiescence as a result of host defence adaptation. Before being tamed, a new or newly reactivated retrotransposon can undergo massive copy number amplification. For instance, more than one million copies of the primate-specific Short Interspersed Element (SINE) *Alu* comprise 11% of the human genome [3]. Even more impressively, approximately 500,000 copies of a single retrotransposon superfamily, *Gypsy*, occupy nearly half of the maize genome [4]. Thus, retrotransposons can overrun a genome within a brief evolutionary period, making their suppression a high host priority.

**Figure 1:**
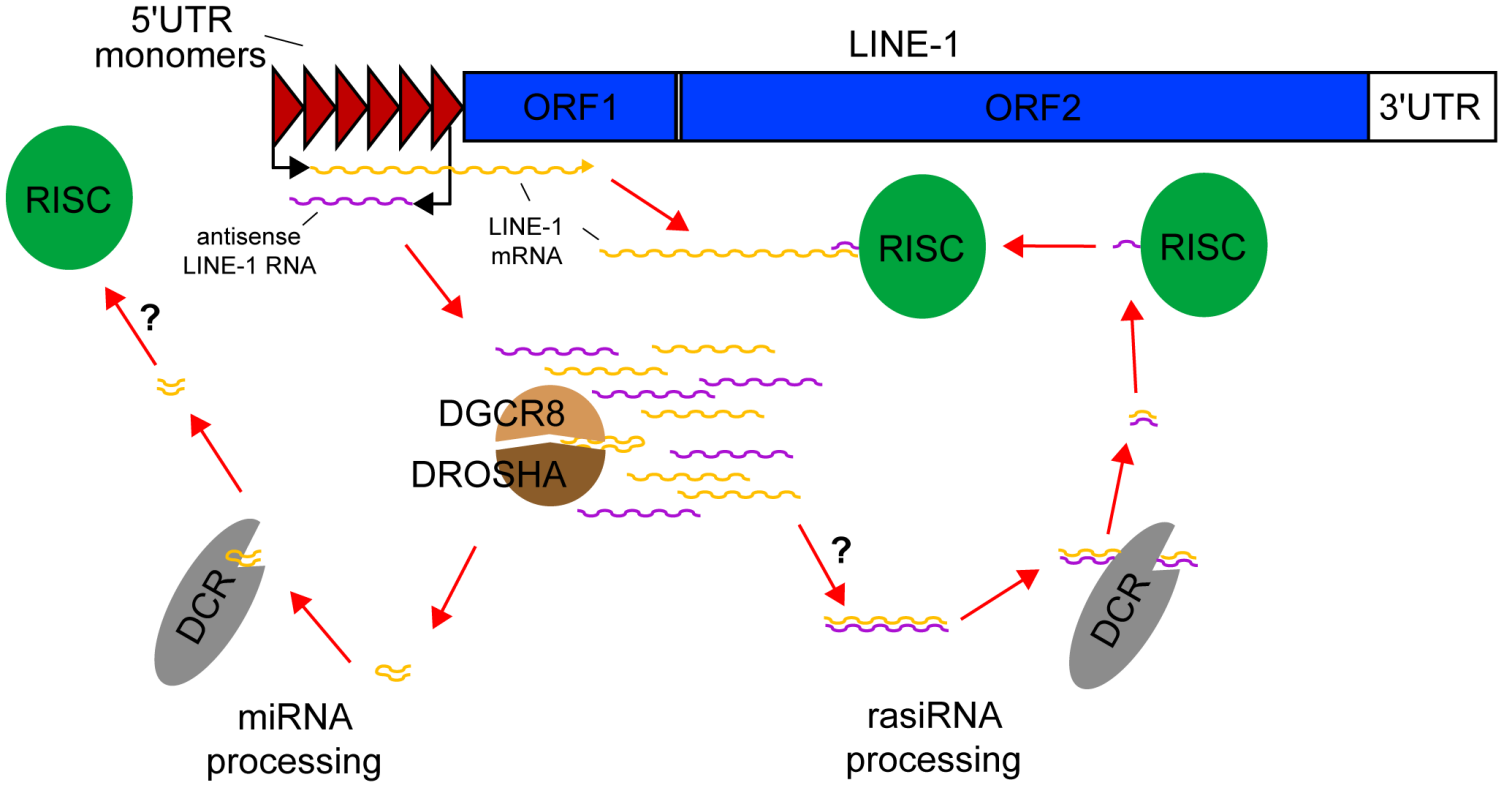
rasiRNAs inhibit LINE-1 expression in mESCs. Mouse LINE-1s are comprised of two ORFs flanked by 5′ and 3′UTRs. Several monomers in the 5′UTR provide promoter activity. Following the LINE-1 expression and copy number variation data of Ciaudo et al., bidirectional transcription of the 5′UTR generates sense and antisense LINE-1 RNAs. The *Drosha*-DGCR8 Microprocessor cleaves these precursors into pre-miRNAs, which are processed into miRNAs by *Dicer*, but may not be loaded into the RISC complex. By contrast, double-stranded RNAs potentially formed by sense/antisense pairing of LINE-1 RNAs are also cleaved by *Dicer* but here generate rasiRNAs, loaded into the RISC complex, which degrade canonical LINE-1 mRNAs. *Dicer* also appears to mediate LINE-1 promoter methylation (not shown).

Retrotransposition requires transcription of an RNA template for DNA-primed reverse transcription. Several cellular defence mechanisms have evolved to hinder this process, including: 1) promoter methylation and heterochromatinisation, 2) degradation of retrotransposon transcripts via RNA interference (RNAi), and 3) host factor prevention or destabilisation of reverse transcription. To describe in detail just one of a myriad of specific inhibitory pathways, repeat associated small interfering RNAs (rasiRNAs) are present in plant, worm, fly, fish, and mouse gametes and, therefore, represent a highly conserved defence against germ line retrotransposition [5]–[8]. A plausible model of rasiRNA biogenesis involves bidirectional transcription of opposed retrotransposon promoters [9], [10], resulting in the formation of double-stranded RNAs (Figure 1). These are cleaved by *Dicer* (DCR) and then assembled with *Argonaute* (AGO) and other proteins into the RNA-induced silencing complex (RISC) that, in turn, produces RNAi against retrotransposon transcripts [11]. The suppressive influence of rasiRNAs, in concert with other pathways, may explain why retrotransposition is more common during embryogenesis than in gametes [12], [13]. Importantly, although rasiRNAs have been found in stem cells and soma, their capacity to suppress retrotransposition during development is relatively unexplored [14]–[16].

In this issue of *PLOS Genetics*, Ciaudo et al. [17] describe rasiRNA-mediated suppression of LINE-1 activity in mouse embryonic stem cells (mESCs). Focusing on the L1-Tf subfamily, where they previously described an unusual rasiRNA signature mapping to the 5′UTR [15], Ciaudo et al. observed that knock-out of *Dicer* markedly decreases L1-Tf promoter methylation and increases L1-Tf transcription, translation, and copy number in cultured mESCs. In particular, DCR^−/−^ mESCs accumulate a remarkable 860 L1-Tf copies (greater than five megabases of genomic DNA) per cell over 20 passages, versus 255 copies per cell in DCR^Flx/Flx^ controls, based on SYBR-Green qPCR targeting the L1-Tf 5′UTR. High-throughput small RNA sequencing then confirmed that DCR^−/−^ mESCs were depleted of approximately 22 nt molecules found in wild-type mESCs, immunoprecipitated with AGO2 and aligned to L1-Tf, and therefore resembling rasiRNAs. Hence, LINE-1 activation in DCR^−/−^ mESCs coincides with rasiRNA depletion and is also possibly influenced by ablation of *Dicer*-mediated LINE-1 promoter methylation.

Intriguingly, a second class of *Dicer*- and AGO2-independent small RNAs were found to “paint” the L1-Tf 5′UTR. Again, assessing L1-Tf transcription and copy number, Ciaudo et al. found that deletion of XRN2 and DGCR8, respective members of the RNA surveillance and *Drosha*-DGCR8 Microprocessor pathways, led to increased L1-Tf transcription but not copy number amplification. These observations agree with other recent reports of small RNAs immunoprecipitated with DGCR8 and enriched for LINE-1 sequences [18], as well as evidence of elevated L1-Tf expression in DGCR8^−/−^ mESCs [19]. As a final experiment, Ciaudo et al. complemented DCR^−/−^ mESCs with human *Dicer* and found that these cells recapitulated wild-type mESC LINE-1 suppression and differentiated normally, unlike DCR^−/−^ mESCs.

Evidence for a reciprocal relationship between rasiRNA depletion and LINE-1 activation significantly advances our understanding of RNAi-mediated control of retrotransposition during mammalian embryogenesis. These data are also important because they address a longstanding question of why rasiRNAs cannot be consistently detected in mammalian somatic cells: small RNAs generated by RNA surveillance and the Microprocessor may cleave the same pool of precursor LINE-1 mRNAs processed by *Dicer* and obscure rasiRNA detection (Figure 1). As Ciaudo et al. note, it is possible that insertional mutagenesis caused by LINE-1 contributes to the reported differentiation defects for DCR^−/−^ mESCs [20], though it is unclear why lesser but still substantial LINE-1 activity is tolerated by wild-type mESCs. Interestingly, experiments using engineered LINE-1 reporters have shown elsewhere [16], [19] that mutation of *Dicer* or the Microprocessor increases LINE-1 mobilisation in cancer cells, with the latter result at odds with data generated here from mESCs. Future advances in high-throughput sequencing and single cell genomics should enable characterisation of endogenous LINE-1 mobilisation events in stem cells and further delineate the multifaceted roles of *Dicer* and other factors in LINE-1 inhibition.
